# Genome-Wide Identification and Characterization of the Annexin (ANN) Gene Family in Potato (*Solanum tuberosum* L.) and Its Role in Salt Stress Response Revealed by Integrated Transcriptome and WGCNA Analysis

**DOI:** 10.3390/genes17091094

**Published:** 2026-09-11

**Authors:** Qingsheng Zhou, Jingchong Li

**Affiliations:** 1 School of Geography and Tourism, Zhengzhou Normal University, Zhengzhou 450044, China; 2 School of Plant Protection and Environment, Henan Institute of Science and Technology, Xinxiang 453000, China

**Keywords:** potato, salt stress, annexin, WGCNA, transcriptome

## Abstract

**Background/Objectives:** Soil salinization threatens global potato production, yet the molecular mechanisms underlying salt tolerance remain poorly understood. This study aimed to elucidate dynamic transcriptomic responses to salt stress, identify key regulatory genes, and characterize the corresponding gene family and its stress-responsive roles. **Methods:** Potato cv. Atlantic plantlets were subjected to 150 mM NaCl stress, with samples collected at 0, 3, 12, 24, and 48 h. Physiological and transcriptomic analyses were performed. WGCNA was employed to identify salt-responsive modules. Genome-wide identification, phylogenetic analysis, gene structure characterization, conserved motif analysis, and promoter cis-element prediction of the annexin gene family were conducted. **Results:** Transcriptome sequencing identified 1394 differentially expressed genes (DEGs) that were consistently responsive to salt stress, with functional enrichment highlighting stress signaling, hormone transduction, and phenylpropanoid biosynthesis pathways. Weighted gene co-expression network analysis (WGCNA) revealed the MEblack module as the key salt-responsive regulatory module, in which annexin genes exhibited high intramodular connectivity. Genome-wide identification uncovered nine annexin genes (*StANN1*–*StANN9*), characterized by uneven chromosomal distribution, conserved exon–intron structures and annexin repeat domains, and expansion through tandem, proximal and segmental duplication under purifying selection. Promoter analysis revealed abundant stress-responsive cis-elements, including ABRE and DRE/CRT motifs, in salt-inducible annexins. Expression profiling demonstrated that *StANN5* and *StANN6* were continuously upregulated under prolonged salt stress, whereas *StANN2* and *StANN3* showed transient early induction. **Conclusions:** These findings establish the central role of annexins in calcium signaling, redox homeostasis, and hormone-mediated stress adaptation, providing candidate targets for molecular breeding of salt-tolerant potato varieties.

## 1. Introduction

Soil salinization has become one of the most severe abiotic stresses threatening global agricultural productivity and food security. According to the Food and Agriculture Organization (FAO), approximately 1.4 billion hectares of land worldwide are currently affected by salinization, while an additional 1 billion hectares are at risk due to climate change and unsustainable agricultural practices [1]. Salt stress restricts plant growth through two primary mechanisms: osmotic stress, which reduces cellular water uptake, and ion toxicity resulting from excessive Na^+^ accumulation that disrupts intracellular ion homeostasis [2]. Additionally, saline conditions trigger excessive production of reactive oxygen species (ROS), which cause oxidative damage but also function as important signaling molecules in stress adaptation [3]. To survive in saline environments, plants have evolved complex regulatory networks encompassing osmotic adjustment, ion transport and compartmentalization, antioxidant defense, hormonal regulation, calcium signaling, and transcriptional reprogramming [4].

Potato (*Solanum tuberosum* L.) is the world’s third most important food crop, with an annual production exceeding 375 million tons [5]. Owing to its high nutritional value, efficient water-use efficiency, and broad environmental adaptability, potato has been recognized by the FAO as a strategic crop for ensuring global food security [6]. Nevertheless, potato is highly sensitive to salt stress, with both vegetative growth and tuber development being severely impaired under saline conditions [7]. Salt stress adversely affects photosynthesis, nutrient acquisition, and phytohormone homeostasis, ultimately leading to substantial yield losses [8]. Physiologically, potato plants respond by enhancing antioxidant enzyme activities, including superoxide dismutase (SOD), catalase (CAT), and peroxidase (POD), concomitant with the accumulation of compatible osmolytes such as proline and soluble sugars [7,8]. At the molecular level, numerous salt-responsive genes associated with ion transport, including NHX, HKT, and components of the SOS signaling pathway, as well as transcription factors from the AP2/ERF, bZIP, and NAC families, have been reported to contribute to salt tolerance [7]. However, these studies have primarily focused on classical ion transporters and transcriptional regulators, while the upstream regulatory components coordinating calcium signaling, ROS homeostasis, and multiple stress-response pathways remain insufficiently understood. Consequently, the key regulatory genes underlying potato salt tolerance remain to be comprehensively identified.

Transcriptome sequencing (RNA-seq) has become an indispensable approach for elucidating molecular responses to abiotic stresses at the genome-wide level. Beyond conventional differential expression analysis, weighted gene co-expression network analysis (WGCNA) enables the identification of highly correlated gene modules and hub genes that orchestrate complex biological processes [9]. As WGCNA considers gene co-expression patterns across the entire transcriptome, it can uncover critical regulatory genes that may not exhibit the largest expression changes but occupy central positions within stress-responsive regulatory networks [10]. This strategy has been successfully applied to identify salt-responsive modules and hub genes in several plant species, including tobacco and cotton, revealing regulatory networks associated with phytohormone signaling, calcium signaling, and MAPK cascades [11,12]. Therefore, integrating transcriptome analysis with WGCNA provides an effective strategy for uncovering novel regulatory genes that govern salt tolerance in potato.

Among the potential regulators involved in plant adaptation to salinity, annexins (ANNs) have attracted increasing attention because of their multifunctional roles in calcium-dependent signaling and cellular stress responses. Annexins constitute a conserved multigene family of calcium-dependent phospholipid-binding proteins widely distributed in eukaryotes [13]. In plants, annexins function not only as membrane-associated proteins but also as important regulators that integrate calcium signaling, ROS homeostasis, ion transport, and hormone-mediated stress responses [14]. Increasing evidence indicates that plant annexins participate in responses to drought, salinity, cold, and oxidative stresses by modulating cytosolic Ca^2+^ influx, maintaining membrane integrity, regulating antioxidant systems, and coordinating downstream stress signaling pathways [15,16]. For example, Arabidopsis annexin 1 (*AtANN1*) functions as a ROS-activated plasma membrane Ca^2+^ channel mediating cytosolic Ca^2+^ accumulation under salt stress and initiates downstream signaling pathways [15]. In rice, *OsANN3* promotes stomatal closure and ABA accumulation to enhance drought tolerance [17], whereas *OsANN1* modulates H_2_O_2_ accumulation to improve thermotolerance [16]. In tomato, overexpression of *AnnSp2* enhances drought and salt tolerance through ABA synthesis and ROS scavenging [18], and in cotton, *GhANN1* regulates ABA biosynthesis, ion homeostasis, and the phenylpropanoid pathway to modulate salt tolerance [19]. In potato, *StANN1* promotes drought tolerance and alleviates photoinhibition [20]. Despite these advances, the annexin gene family has not been systematically characterized in potato, and its potential roles in salt stress perception, signal transduction, and adaptive regulation remain largely unknown.

In the present study, we combined physiological analyses with transcriptome sequencing to investigate the dynamic responses of potato to salt stress. WGCNA was subsequently employed to identify key regulatory modules and candidate hub genes associated with salt tolerance. Among the candidate genes, members of the annexin family were identified as highly connected components of the salt-responsive co-expression network, highlighting their potential regulatory importance. We therefore performed a genome-wide identification and characterization of the potato annexin gene family, encompassing phylogenetic relationships, gene structure, conserved motifs, cis-regulatory elements, chromosomal distribution, and expression patterns under salt stress. Collectively, this study provides new insights into the molecular mechanisms underlying potato salt tolerance and lays a theoretical foundation for the genetic improvement of salt-tolerant potato cultivars.

## 2. Materials and Methods

### 2.1. Plant Cultivation

Aseptic plantlets of potato (*S. tuberosum* L.) cv. Atlantic were used as plant materials. The plantlets were initially propagated and maintained aseptically on solid half-strength Murashige and Skoog (1/2 MS) medium under controlled growth conditions. Uniform plantlets at similar developmental stages were selected, gently washed with deionized water to remove residual MS medium, and transferred to a hydroponic system containing full-strength Hoagland nutrient solution for a 3-day acclimation period. During acclimation, plantlets were monitored daily for visible signs of transplant stress, including leaf wilting, chlorosis, or root browning. No stress symptoms were observed after 24 h, and by 72 h all plantlets exhibited fully turgid leaves, healthy white root tips, and normal incremental growth, indicating successful adaptation to the liquid culture environment. Following acclimation, salt stress was imposed by supplementing the Hoagland solution with 150 mM NaCl. Leaf tissues were collected at five time points: 0 h (control, without NaCl treatment), 3 h, 12 h, 24 h, and 48 h after salt stress exposure. All hydroponic cultures and stress treatments were conducted in a controlled growth chamber. For each time point, three independent hydroponic containers were established as biological replicates (*n* = 3 per time point, 15 containers in total). Each container held 6 plantlets. At sampling, two randomly selected plantlets per container were harvested, and their leaf tissues were pooled to constitute one biological replicate. Each container was used only once. Collected samples were immediately frozen in liquid nitrogen and stored at −80 °C pending physiological parameter measurements and transcriptome sequencing.

### 2.2. Transcriptome Sequencing

Total RNA was extracted from collected samples using the MJZol Total RNA Extraction Kit (Shanghai Meiji Biotechnology Co., Ltd., Shanghai, China). RNA quality and quantity were assessed by agarose gel electrophoresis and spectrophotometric analysis using a NanoDrop 2000 system (NanoDrop Technologies, Wilmington, DE, USA). RNA integrity was further evaluated using an Agilent5300 Bioanalyzer (Agilent Technologies, Santa Clara, CA, USA). Sequencing libraries were prepared using the Illumina Stranded mRNAPrep Kit (Illumina Inc., San Diego, CA, USA) according to the manufacturer’s instructions, and 150 bp paired-end sequencing was performed on an Illumina NovaSeq X Plus platform (Illumina Inc., San Diego, CA, USA).

After quality filtering and adapter trimming, clean reads were aligned to the potato reference genome using HISAT2 (http://ccb.jhu.edu/software/hisat2/index.shtml, accessed 3 April 2026). The reference genome used was SolTub_3.0 (GCA_000226075.1, Ensembl Plants). Transcript abundance was quantified using RSEM, and differentially expressed genes (DEGs) were identified using DESeq2. Genes with an adjusted *p*-value (FDR) < 0.05 and |log_2_(fold change)| ≥ 1 were considered significantly differentially expressed.

### 2.3. GO and KEGG Enrichment Analysis

To functionally characterize the identified differentially expressed genes (DEGs), GO enrichment analysis was performed using GOATOOLS based on over-representation analysis. KEGG pathway enrichment analysis was conducted using a hypergeometric test implemented with the SciPy package in Python 3.12. The Benjamini–Hochberg procedure was applied to correct for multiple testing, and pathways with adjusted *p*-values (FDR) < 0.05 were considered significantly enriched.

### 2.4. Quantitative Real-Time PCR (qRT-PCR)

Reverse transcription was performed using HiScript II Q RT SuperMix (+gDNA wiper; Vazyme, Nanjing, China) according to the manufacturer’s instructions for first-strand cDNA synthesis. Quantitative real-time PCR was conducted using a Roche LightCycler 480 II Real-Time PCR System (Roche Diagnostics, Mannheim, Germany). Relative transcript abundance was calculated using the comparative 2^−ΔΔCt^ method [21] and normalized against *EF1A* as the internal reference gene. RNA-seq data confirmed that *EF1A* was stably expressed under salt stress (FPKM CV < 10% across all time points), supporting its use as the internal reference gene. The RNA samples used for qRT-PCR validation were the same as those used for RNA-seq analysis. Primer sequences are listed in Table S1.

### 2.5. Measurement of Physiological Parameters

To evaluate the physiological responses of plants to salt stress, six physiological parameters were measured, including proline (Pro) content, H_2_O_2_ content, MDA content, and the activities of SOD, CAT, and POD. All physiological measurements were conducted with three independent biological replicates.

The acid ninhydrin method was employed for Pro quantification. Briefly, 0.1 g of fresh leaf tissue was homogenized into a fine powder in liquid nitrogen and extracted with 1 mL of 80% ethanol at 100 °C for 15 min. After cooling, the extracts were centrifuged at 10,000× *g* for 10 min, and the supernatants were collected. Subsequently, 0.1 mL of the extract was mixed with 0.1 mL of acid ninhydrin reagent and 0.1 mL of glacial acetic acid, followed by incubation at 100 °C for 30 min. After cooling, 0.5 mL of toluene was added, and the mixture was vigorously vortexed and allowed to stand for 2 h to achieve complete phase separation. The absorbance of the upper toluene phase was measured at 520 nm using a spectrophotometer. Proline concentration was calculated based on a standard curve and expressed as μg g^−1^ fresh weight (FW).

H_2_O_2_ content was quantified using the ferric oxidation-xylenol orange (FOX) assay. Briefly, 0.1 g of fresh leaf tissue was ground in liquid nitrogen and extracted with 1 mL of pre-chilled acetone. The homogenate was centrifuged at 10,000× *g* for 15 min at 4 °C, and the supernatant was collected for analysis. For the colorimetric reaction, 150 μL of extract was mixed with 100 μL of distilled water, 250 μL of 0.8 mM ferrous ammonium sulfate, 250 μL of 400 mM sorbitol, and 250 μL of 0.4 mM xylenol orange. The reaction mixture was incubated at 30 °C for 30 min, and absorbance was measured at 560 nm. H_2_O_2_ content was calculated using a standard curve prepared with serial dilutions of a 30% H_2_O_2_ stock solution.

MDA content was determined using the thiobarbituric acid (TBA) reaction method. Briefly, 0.1 g of fresh leaf tissue was homogenized in liquid nitrogen and extracted with 1 mL of 10% trichloroacetic acid (TCA). After centrifugation at 10,000× *g* for 10 min, the supernatant was mixed with an equal volume of 0.6% TBA solution. The reaction mixture was heated in a boiling water bath for 20 min and subsequently cooled to 25 °C. Absorbance values were recorded at 450, 532, and 600 nm. MDA content was calculated according to the method described by Wang et al. [22].

For antioxidant enzyme activity assays, crude enzyme extracts were prepared as follows. Approximately 0.1 g of fresh leaf tissue was ground into powder in liquid nitrogen and homogenized in 1 mL of 0.1 M phosphate buffer (pH 7.5) containing 1 mM EDTA. The homogenates were centrifuged at 15,000× *g* for 20 min at 4 °C, and the supernatants were collected for enzyme activity measurements.

SOD (EC 1.15.1.1) activity was determined using the nitroblue tetrazolium (NBT) photoreduction inhibition assay according to Zhang et al. [23]. Absorbance was measured at 560 nm. One unit of SOD activity was defined as the amount of enzyme required to inhibit 50% of NBT photoreduction, and enzyme activity was expressed as U g^−1^ FW.

CAT (EC 1.11.1.6) activity was determined by monitoring the decrease in absorbance at 240 nm caused by H_2_O_2_ decomposition. The 1 mL reaction mixture contained 0.5 mL of enzyme extract, 0.35 mL of 100 mM phosphate buffer (pH 7.0), and 0.15 mL of 100 mM H_2_O_2_. CAT activity was calculated based on the rate of H_2_O_2_ consumption and expressed as U g^−1^ min ^−1^ FW.

POD (EC 1.11.1.7) activity was measured using the guaiacol oxidation method according to Polle et al. [24]. The reaction mixture consisted of 0.75 mL of 1.92 mM guaiacol, 0.2 mL of 50 mM H_2_O_2_, and 0.05 mL of enzyme extract. The increase in absorbance was monitored at 470 nm.

For CAT and POD assays, one unit of enzyme activity was defined as a 0.01 increase in absorbance per minute and expressed as U g^−1^ min^−1^ FW.

### 2.6. Weighted Gene Co-Expression Network Analysis (WGCNA)

WGCNA was performed using the WGCNA R package (v1.51). DEGs responsive to salt stress with FPKM > 1 under control conditions were selected for network construction. The soft-thresholding power was determined as β = 12 based on the scale-free topology criterion (R^2^ > 0.85), while the remaining parameters were set to their default values. Gene co-expression networks were visualized using Cytoscape (v3.10.0). To focus on the core salt-responsive transcriptional network underlying sustained stress adaptation, WGCNA was performed using 1179 DEGs that were differentially expressed at all four time points (3, 12, 24, and 48 h) under salt stress (FPKM ≥ 1 under control conditions). This pre-filtering strategy, which has been widely employed in plant stress transcriptomics to reduce noise and prioritize condition-specific co-expression networks [25], is designed to capture genes involved in prolonged transcriptional reprogramming.

### 2.7. Bioinformatics Analysis

Genome assemblies of *S. tuberosum*, *Oryza sativa*, *Arabidopsis thaliana*, *Brassica oleracea*, *Solanum lycopersicum*, and *Medicago truncatula* were downloaded from Ensembl Plants (https://plants.ensembl.org/, accessed 3 April 2026). To facilitate subsequent hidden Markov model (HMM)-based identification, a local protein sequence database was constructed on a Bio-Linux virtual machine.

The hidden Markov model (HMM) profile of the annexin domain (PF00191) was retrieved from the Pfam database (http://pfam.xfam.org, accessed 3 April 2026). Sequences containing the conserved annexin domain were identified using HMMER (v3.3.2) and retained for further analysis. Multiple sequence alignment of ANN proteins from *S. tuberosum* and their homologs from *A. thaliana*, *O. sativa*, *B. oleracea*, *S. lycopersicum*, and *M. truncatula* was performed using ClustalX v2.1. A phylogenetic tree was constructed using the Maximum Likelihood (ML) method implemented in MEGA12, with 1000 bootstrap replicates to evaluate branch support [26].

The physicochemical properties of StANN proteins, including amino acid length, molecular weight (MW), and theoretical isoelectric point (pI), were predicted using EMBOSS Pepstats (https://www.ebi.ac.uk/Tools/seqstats/emboss_pepstats/, accessed 5 April 2026). Subcellular localization was predicted using WoLF PSORT (https://wolfpsort.hgc.jp/, accessed 5 April 2026). Chromosomal locations of the StANN genes were visualized using MapGene2Chromweb v2.0 (http://mg2c.iask.in/mg2c_v2.0, accessed 5 April 2026).

To identify duplicated StANN genes, the coding sequences (CDSs) of all family members were used to construct a local BLAST database. Pairwise sequence alignments were performed using BLASTN (v2.15.0) with an E-value threshold of 1 × 10^−20^. Gene pairs sharing at least 75% nucleotide sequence identity and an alignment length covering at least 75%of the longer coding sequence were considered duplicated gene pairs. Duplicated pairs were classified based on chromosomal location and physical distance: tandem duplication (same chromosome, <100 kb, <5 intervening genes); proximal duplication (same chromosome, >100 kb); and segmental duplication (different chromosomes) [27].

For promoter analysis, 1.5 kb genomic sequences upstream of the translation initiation codon (ATG) for each StANN gene were extracted using an in-house Perl script running on the Bio-Linux platform. Cis-acting regulatory elements within the promoter regions were identified using the PlantCARE database (http://bioinformatics.psb.ugent.be/webtools/plantcare/html/, accessed 5 April 2026).

### 2.8. Statistical Analysis

All physiological data were presented as the mean ± standard deviation (SD) of three independent biological replicates. Statistical analyses were performed using SPSS 22.0 (IBM Corp., Armonk, NY, USA), using one-way analysis of variance (ANOVA), followed by Duncan’s multiple range test (DMRT) for multiple comparisons. Data were checked for normality and variance homogeneity prior to ANOVA (*p* > 0.05; Table S7). Differences were considered statistically significant at *p* < 0.05. Data visualization was performed using Origin 2020 (OriginLab Corporation, Northampton, MA, USA).

## 3. Results

### 3.1. Transcriptomic Profiling of Salt Stress-Responsive Genes

To elucidate the dynamic gene expression patterns underlying salt stress responses in potato, we performed transcriptome sequencing. The sequencing data were of high quality, with Q20 and Q30 values exceeding 98.5% and 96.6%, respectively, and 83.97–85.13% of reads mapping to the reference genome (Tables S2 and S3). Pearson correlation and PCA analyses confirmed high reproducibility among biological replicates and clear temporal separation across time points (Figure S1). Notably, the three biological replicates at 0 h (post-acclimation control) clustered tightly together without dispersion, indicating a stable transcriptomic baseline prior to stress initiation. The 0 h group was distinctly separated from all salt-treated time points, confirming that the observed temporal shifts were driven by NaCl exposure rather than by pre-existing variation or residual transplant effects. Collectively, these results demonstrate the reliability of the dataset and its suitability for subsequent differential expression analysis. To validate the reliability of the transcriptome data, qRT-PCR was conducted on ten representative genes, and a linear regression analysis was performed between the expression levels obtained from both methods, with the results presented in scatter plots (Figure S2). A significant linear correlation was observed between the two datasets, indicating a high degree of credibility for the transcriptome sequencing results.

**Figure 1:**
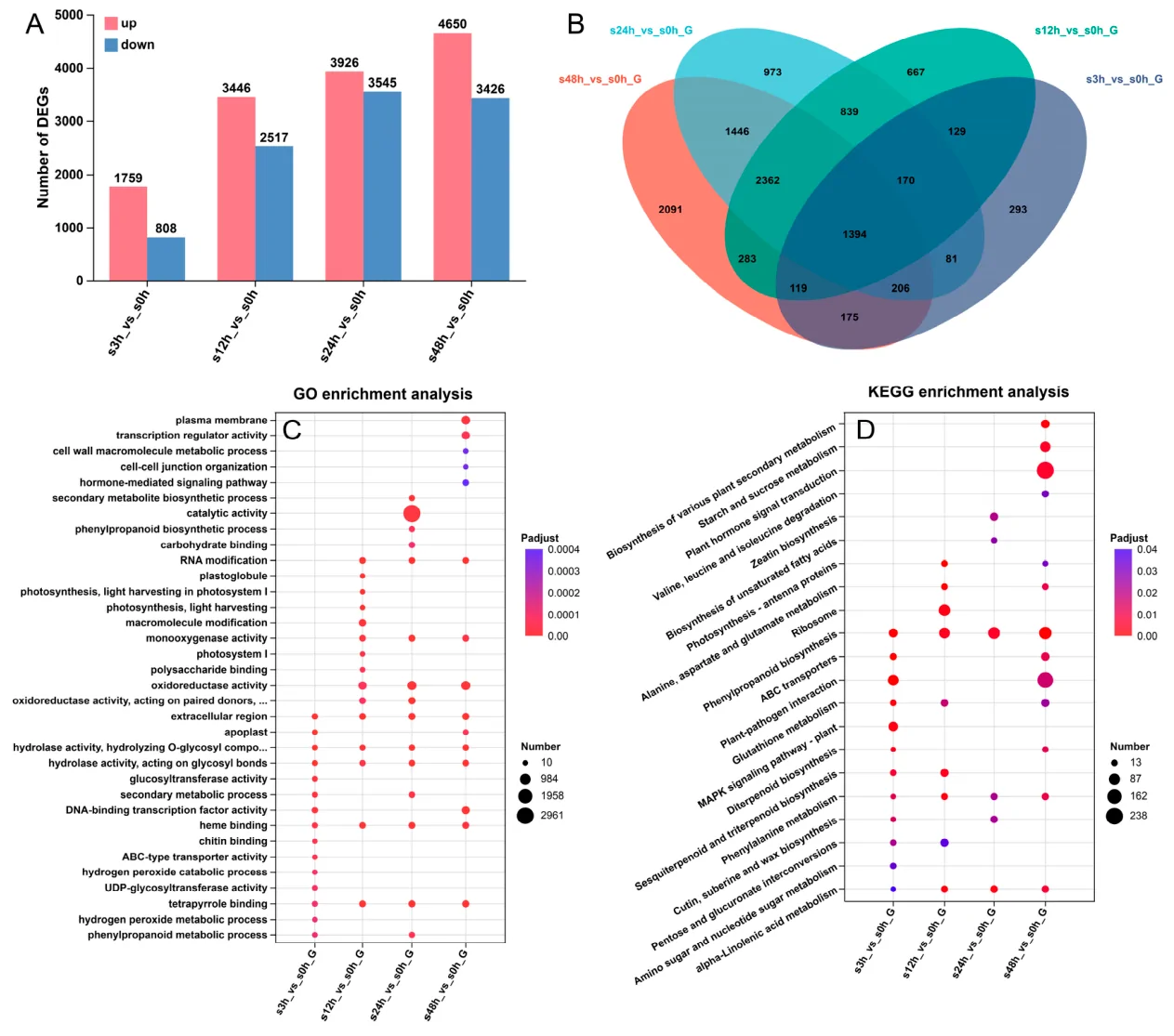
Dynamic transcriptomic analysis of potato under salt stress. (**A**) Number of upregulated and downregulated DEGs at 3, 12, 24, and 48 h post-treatment. (**B**) Venn diagram showing overlapping DEGs across four time points. (**C**) GO enrichment analysis. (**D**) KEGG pathway enrichment analysis.

Differential expression analysis showed a progressive increase in DEGs during salt stress, with the number of upregulated genes increasing markedly from 1759 at 3 h to 4650 at 48 h, while downregulated genes also increased (Figure 1A). Venn analysis identified 1394 genes that were differentially expressed at all four time points, suggesting a core set of salt-responsive genes (Figure 1B).

GO enrichment analysis showed that DEGs at different stages were enriched in distinct GO terms, reflecting stage-specific transcriptional reprogramming during salt stress (Figure 1C). Several GO terms, however, were significantly enriched at multiple time points, including oxidoreductase activity, monooxygenase activity, and hydrolase activity, indicating the activation of conserved defense mechanisms (Figure 1C). Notably, oxidoreductase activity was significantly enriched from 12 h onward, highlighting the importance of redox homeostasis in sustained stress adaptation. Catalytic activity was most prominently enriched at 24 h, with the largest number of associated genes, indicating extensive enzymatic activation at this critical transition stage.

KEGG enrichment analysis further showed that potato plants modulate distinct metabolic pathways at different stages of salt stress, whereas several pathways were consistently enriched across multiple time points (Figure 1D). Notably, plant hormone signal transduction, phenylpropanoid biosynthesis, glutathione metabolism, and the biosynthesis of various plant secondary metabolites were enriched, underscoring their central roles in coordinating the salt stress response. In addition, starch and sucrose metabolism and photosynthesis–antenna proteins were prominently enriched at specific stages, reflecting metabolic reprogramming to maintain energy balance and osmotic homeostasis under salinity.

### 3.2. WGCNA-Based Dissection of Salt Stress-Responsive Gene Modules and Hub Genes

**Figure 2:**
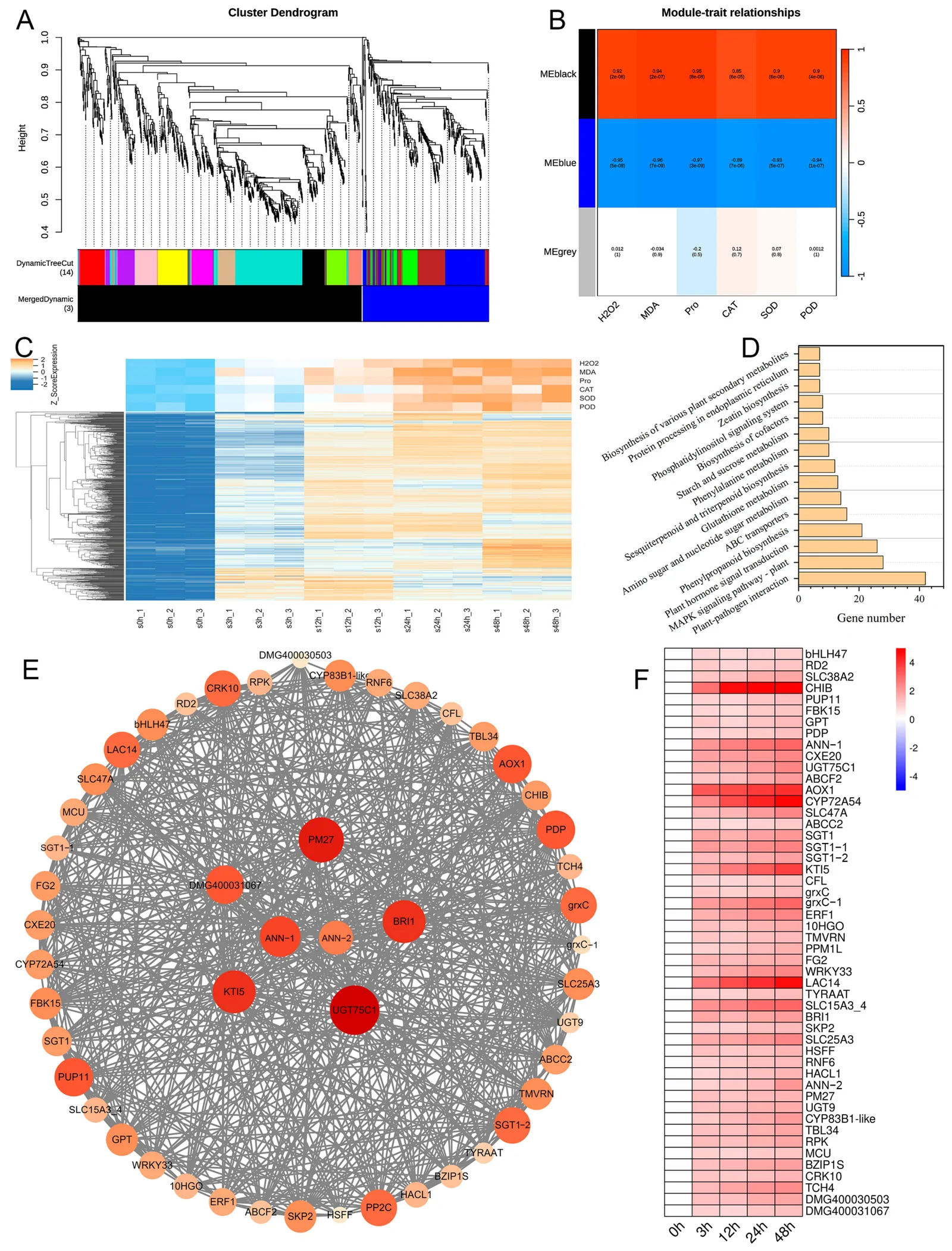
WGCNA-based identification of salt stress-responsive gene modules and hub genes in potato. (**A**) Hierarchical clustering dendrogram of 1179 DEGs (FPKM ≥ 1) and dynamic branch cutting. (**B**) Module–trait correlation heatmap. (**C**) Expression heatmap of genes within the MEblack module across all samples. (**D**) KEGG pathway enrichment analysis of MEblack module genes. (**E**) Co-expression network of MEblack module hub genes. Node size and color intensity reflect connectivity degree; larger and darker red nodes indicate higher connectivity. (**F**) Temporal expression profiles of selected hub genes across five time points (0, 3, 12, 24, and 48 h). Expression values are Z-score normalized.

To investigate co-expression patterns among salt stress-responsive genes and their relationships with physiological alterations, WGCNA was performed using 1179 genes that were differentially expressed across all four time points (FPKM ≥ 1), together with six physiological parameters, including Pro, MDA, H_2_O_2_, SOD, CAT, and POD (Figure S3). Hierarchical clustering initially classified these genes into 14 modules, which were subsequently merged into three major modules (MEblack, MEblue, and MEgrey) based on module similarity (Figure 2A). Module–trait correlation analysis demonstrated that the MEblack module showed the strongest positive correlations with all six physiological indices, particularly Pro (r = 0.95, *p* = 8 × 10^−6^), MDA (r = 0.94, *p* = 2 × 10^−7^), and H_2_O_2_ (r = 0.92, *p* = 2 × 10^−6^), highlighting its close association with physiological responses under salt stress (Figure 2B). Therefore, the MEblack module was selected for further analysis.

Expression patterns of genes within the MEblack module showed a gradual increase during salt stress treatment, with transcript abundance markedly elevated from 3 h and reaching the highest levels at 48 h (Figure 2C), indicating continuous activation of this gene set during prolonged salt exposure. KEGG enrichment analysis revealed that MEblack genes were significantly associated with several pathways involved in abiotic stress adaptation, including plant hormone signal transduction, MAPK signaling pathway–plant, phenylpropanoid biosynthesis, glutathione metabolism, and plant–pathogen interaction (Figure 2D). The enrichment of hormone signaling and MAPK-related pathways further highlighted the involvement of regulatory signaling networks in coordinating salt stress responses.

To identify potential regulatory hub genes within the MEblack module, a co-expression network was constructed using genes with module membership (MM) ≥ 0.9 and |gene significance (GS)| ≥ 0.9 (Table S4). It is important to note that hub gene identification in WGCNA is based on intramodular connectivity and gene significance—metrics that reflect topological centrality within the network—rather than on the magnitude or direction of expression changes alone. In the network, node size and colour intensity reflected connectivity degree, with larger and darker red nodes representing higher connectivity (Figure 2E). Several genes showed highly connected network positions, including *UGT75C1*, *PM27*, *KT15*, and *BRI1*, which exhibited the largest node sizes and strongest colour intensity, indicating their high connectivity within the module. In addition, *StANN5* (provisionally annotated as *ANN-1* in the WGCNA screening) and *StANN6* (*ANN-2*) displayed prominent network connectivity among the identified hub genes, suggesting their close association with other salt-responsive genes. While several hub genes, including *UGT75C1*, *PM27*, *KT15*, and *BRI1*, showed higher connectivity degrees (Figure 2E; Table S4), these belong to previously characterized gene families in potato. We therefore selected *StANN5* and *StANN6* as novel candidates for subsequent genome-wide family identification.

### 3.3. Genome-Wide Identification and Evolutionary Analysis of the Annexin Gene Family in Potato

A total of nine annexin genes were identified in the potato genome and designated *StANN1*–*StANN9* according to their chromosomal positions (Table 1). The encoded proteins consisted of 309–325 amino acids, with predicted molecular weights ranging from 35.59 to 36.63 kDa and theoretical isoelectric points (pI) ranging from 5.34 to 9.07. The instability index varied among family members, with *StANN4* showing the highest value (46.39), indicating relatively lower protein stability. All StANN proteins exhibited hydrophilic characteristics, as reflected by their negative GRAVY values (−0.541 to −0.196). Subcellular localization prediction using WoLF PSORT suggested diverse cellular distributions among StANN proteins. For example, *StANN1* was predicted to localize to the cytoplasm, cytoskeleton, and nucleus, whereas *StANN2* exhibited the widest predicted distribution, including the cytoplasm, plastid, chloroplast, nucleus, and mitochondria. In contrast, *StANN9* showed a relatively restricted localization pattern, mainly in the cytoplasm and cytoskeleton.

**Figure 3:**
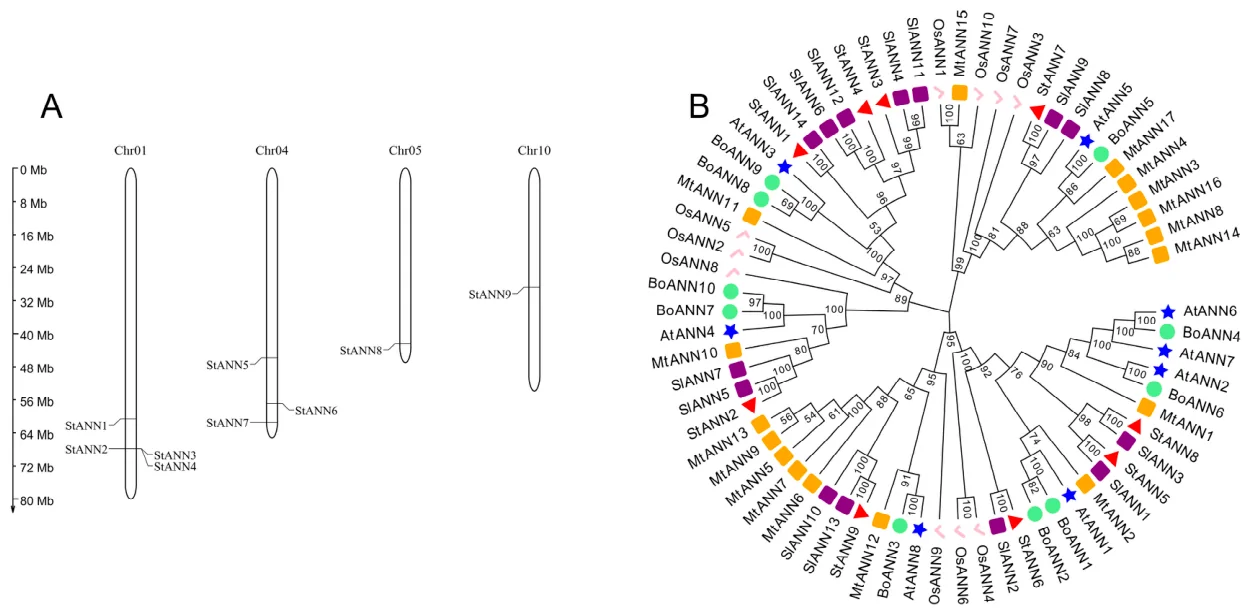
Chromosomal distribution and phylogenetic analysis of the annexin gene family in potato. (**A**) Chromosomal localization of nine StANN genes on potato chromosomes. (**B**) Phylogenetic tree of annexin proteins from potato (*Solanum tuberosum*), Arabidopsis (*Arabidopsis thaliana*), cabbage (*Brassica oleracea*), rice (*Oryza sativa*), Medicago (*Medicago truncatula*), and tomato (*Solanum lycopersicum*). The tree was constructed using the Maximum Likelihood method with 1000 bootstrap replicates. Bootstrap values are indicated at the nodes.

Chromosomal localization analysis revealed an uneven distribution of StANN genes across four chromosomes (Figure 3A). Chromosome 1 contained the largest number of annexin genes, including *StANN1*–*StANN4*, whereas Chr04, Chr05, and Chr10 harbored two, one, and two genes, respectively. The close physical arrangement of *StANN1*–*StANN4* on Chr01 suggested the occurrence of local gene duplication events.

To investigate the evolutionary relationships of annexin proteins, a phylogenetic tree was constructed using 68 annexin sequences from potato, *Arabidopsis*, cabbage (*Brassica oleracea*), rice (*Oryza sativa*), Medicago (*Medicago truncatula*), and tomato (*Solanum lycopersicum*). The annexin proteins were classified into five major clades (Figure 3B). Potato annexins were distributed among all five groups, indicating both evolutionary conservation and diversification within this gene family. Several StANN proteins displayed close evolutionary relationships with annexins from other *Solanaceae* species. Specifically, *StANN5* and *StANN8* clustered with *SlANN1* and *SlANN3* with strong bootstrap support (100%), consistent with the close phylogenetic relationship between potato and tomato. *StANN6* was grouped with *SlANN2*, while *StANN9* showed close associations with *SlANN10* and *SlANN13*, respectively. Meanwhile, *StANN1*, *StANN3*, and *StANN4* occupied independent evolutionary branches, with *StANN3* clustering with *SlANN4* and *SlANN11*, and *StANN4* grouping with *SlANN6* and *SlANN12*.

Duplication analysis identified four duplicated gene pairs, including *StANN1*–*StANN3*, *StANN1*–*StANN4*, *StANN3*–*StANN4*, and *StANN5*–*StANN8* (Figure S4). Among them, the first three pairs were derived from tandem duplication events on Chr01, whereas *StANN5*–*StANN8* resulted from segmental duplication between Chr04 and Chr05. The Ka/Ks ratios of all duplicated gene pairs were below 1 (0.22–0.31), indicating that these duplicated genes have primarily evolved under strong purifying selection (Table S5).

### 3.4. Gene Structure and Conserved Motif Analysis of Potato Annexins

**Figure 4:**
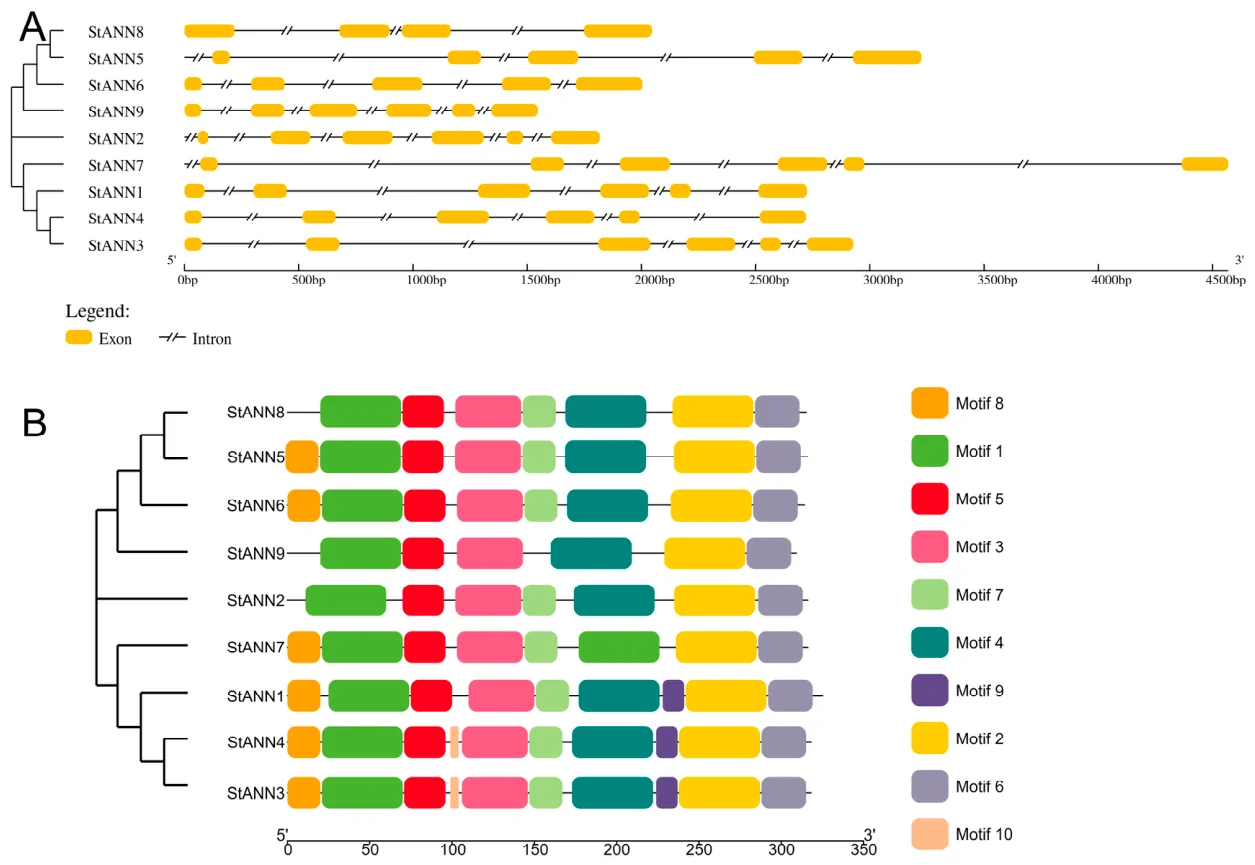
Gene structure and conserved motif analysis of potato annexin genes. (**A**) Exon–intron organization of nine StANN genes. (**B**) Conserved motif distribution in StANN proteins. Ten distinct motifs were identified using MEME.

To gain insights into the structural diversity and evolutionary conservation of potato annexin genes, we analyzed their exon–intron organizations and conserved protein motifs. The gene structure analysis revealed considerable variation in genomic architecture among the nine StANN genes (Figure 4A). The number of exons ranged from four (*StANN8*) to six (*StANN3* and *StANN4*), with most genes containing six exons. Notably, *StANN7* exhibited the most complex structure, featuring six exons and the longest genomic sequence (~4500 bp), whereas *StANN8* displayed the simplest organization, consisting of only four exons. The lengths of the introns varied substantially even among genes with similar exon counts; for instance, both *StANN1* and *StANN9* contained six exons, yet *StANN1* exhibited markedly longer introns. These structural variations suggest that intron expansion and contraction have contributed to the diversification of the annexin family in potatoes.

Conserved motif analysis using MEME identified ten distinct motifs across the StANN proteins (Figure 4B, Table S6). Motifs 1, 2, 3, 5, and 6 were present in all nine members, indicating their fundamental importance for annexin structure and function. Motifs 4 and 7 were detected in most proteins, with the exception of *StANN9*, which lacked Motif 7. Interestingly, Motif 9 was exclusively found in *StANN1*, *StANN3*, and *StANN4*, while Motif 10 was restricted to *StANN3* and *StANN4*, suggesting potential functional specialization among these members. The motif arrangement was highly conserved among phylogenetically closely related proteins; for example, *StANN5* and *StANN6*, as well as *StANN3* and *StANN4*, shared identical motif compositions and orders, consistent with their recent duplication origins. In contrast, *StANN7* exhibited a unique motif pattern, with Motif 4 replaced by Motif 1 in the corresponding position, which may reflect its distinct evolutionary trajectory. Overall, the high conservation of core motifs across all StANN proteins underscores the structural integrity of the annexin domain, while the presence of lineage-specific motifs implies functional diversification within this gene family.

### 3.5. Cis-Acting Element Analysis of StANN Gene Promoters

**Figure 5:**
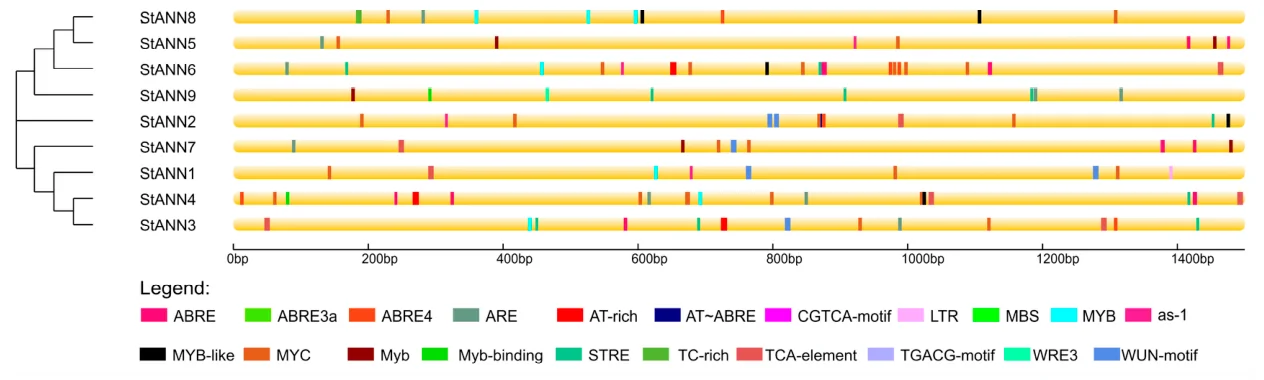
Cis-acting regulatory element analysis of StANN gene promoters. Distribution of stress-responsive cis-acting elements in the 1.5 kb upstream promoter regions of nine StANN genes. Elements were predicted using the PlantCARE database.

To investigate the transcriptional regulatory mechanisms of potato annexin genes under salt stress, we analyzed the 1.5 kb upstream promoter regions of all nine StANN genes using PlantCARE. A diverse array of stress-responsive cis-acting elements was identified (Figure 5), including several directly implicated in abiotic stress responses. The ABRE (abscisic acid-responsive element) and ABRE3a/ABRE4 motifs, known to mediate ABA-dependent signaling pathways, were widely distributed across most StANN promoters, consistent with the central role of ABA in salt stress adaptation. Additionally, ARE (anaerobic-responsive element) and MBS (MYB binding site) were detected in multiple genes, suggesting involvement in hypoxia and drought/salt stress responses, respectively. The WUN-motif (wound-responsive element) and TCA-element were also present, indicating potential cross-talk between biotic and abiotic stress signaling. Notably, *StANN1*, *StANN3*, and *StANN4* harbored the richest complement of stress-responsive elements, implying their significant roles in stress perception. In contrast, *StANN8* and *StANN9* contained relatively fewer regulatory motifs, which may reflect functional divergence. The prevalence of ABA- and MYB-related elements across the StANN family suggests that these transcriptional regulators coordinate annexin-mediated salt stress responses in potatoes.

### 3.6. Tissue-Specific and Salt Stress-Responsive Expression Profiling of StANN Genes

**Figure 6:**
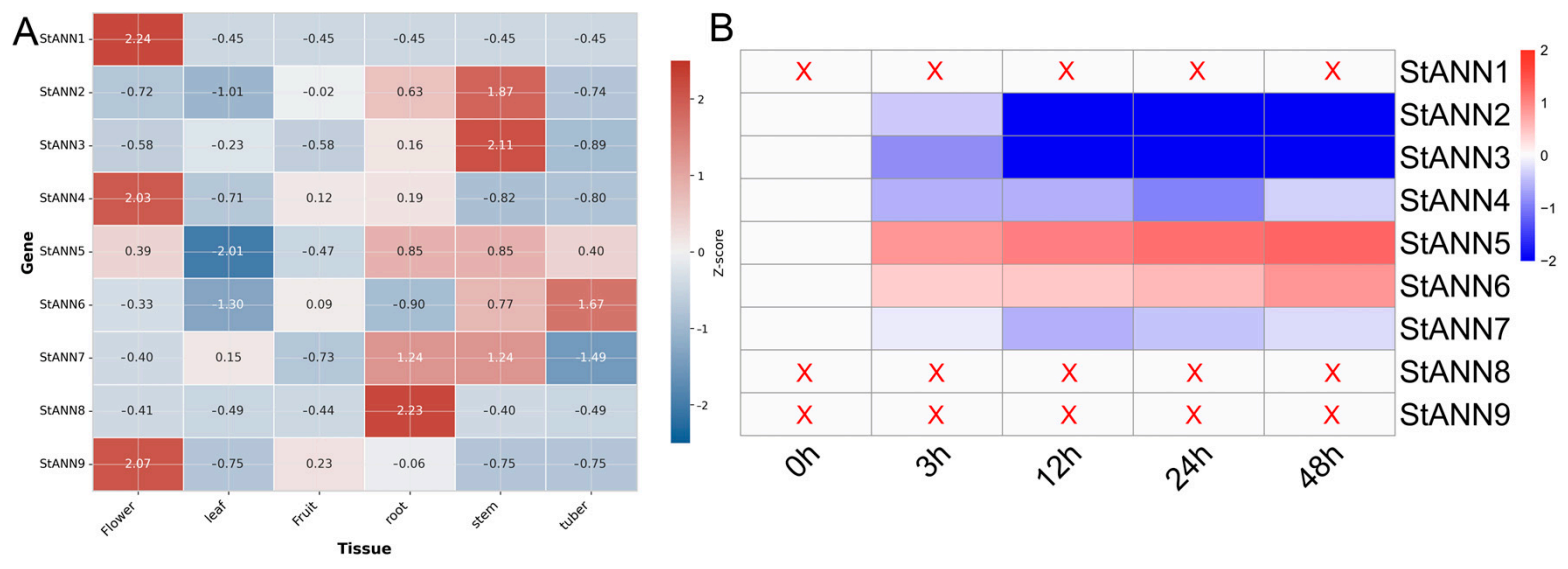
Tissue-specific and salt stress-responsive expression profiling of StANN genes. (**A**) Tissue-specific expression patterns of nine StANN genes across six tissues (flower, leaf, fruit, root, stem, and tuber) based on publicly available RNA-seq data from Spud DB. Expression values are Z-score normalized. (**B**) Dynamic expression changes in StANN genes under 150 mM NaCl stress at 0, 3, 12, 24, and 48 h. Expression values are Z-score normalized. “X” indicates no detectable expression.

To elucidate the functional relevance of potato annexin genes in salt stress adaptation, we first examined their tissue-specific expression patterns utilizing publicly available RNA-seq data from Spud DB. As shown in Figure 6A, the nine StANN genes exhibited distinct yet partially overlapping expression profiles across six different tissues. *StANN1*, *StANN4*, and *StANN9* were predominantly expressed in flowers, with *StANN1* showing the highest enrichment (Z-score = 2.24), suggesting its potential role in reproductive development. Conversely, *StANN2* and *StANN3* displayed preferential expression in stems. Notably, *StANN8* demonstrated root-specific expression (Z-score = 2.23), implicating its involvement in root-mediated stress responses. *StANN5* and *StANN6* exhibited relatively broad expression across leaf, root, and stem tissues, with *StANN6* also enriched in tubers (Z-score = 1.67). The differential tissue distribution of StANN genes implies functional specialization, with root- and stem-preferential members potentially playing more direct roles in sensing and transducing salt stress signals.

We next investigated the dynamic expression changes in StANN genes following a 150 mM NaCl treatment using our transcriptome data. As illustrated in Figure 6B, the salt-responsive expression patterns were categorized into three distinct groups. *StANN5* and *StANN6* were strongly and progressively upregulated from 3 h to 48 h, with expression levels increasing over time, indicating their sustained activation during prolonged salinity exposure. *StANN2* and *StANN3* exhibited specific induction at 3 h but were subsequently downregulated at 12–48 h, suggesting their involvement in early stress signaling. In contrast, *StANN4* and *StANN7* maintained relatively stable or slightly suppressed expression throughout the time course. Strikingly, *StANN1*, *StANN8*, and *StANN9* were not expressed (marked with X) in leaf tissues under either control or salt stress conditions.

## 4. Discussion

### 4.1. Transcriptomic Reprogramming and Salt Stress Response Dynamics

During salt stress treatment, the number of DEGs exhibited a progressive increase, with the number of upregulated genes increasing from 1759 at 3 h to 4650 at 48 h. This pattern suggests that the plant response gradually shifted from early osmotic stress perception to sustained responses associated with ion toxicity and oxidative damage. This temporal dynamic pattern is consistent with the classical “two-phase model” of salt stress responses, in which the rapid osmotic stress phase (0–3 h) primarily triggers early signal transduction events, whereas the subsequent ionic stress phase (12–48 h) is characterized by Na^+^/K^+^ homeostasis disruption, ROS accumulation, metabolic reprogramming, and extensive transcriptional responses [2,28]. Meanwhile, the continuous increases in MDA, H_2_O_2_, and proline contents from 3 h to 48 h further support this hypothesis, indicating that prolonged stress exposure progressively aggravates cellular oxidative damage while simultaneously activating multilayered adaptive regulatory mechanisms. Similar continuous accumulation of DEGs under salt stress has also been reported in time-series transcriptomic studies of cotton [19] and tomato [29], suggesting that this dynamic transcriptional response pattern may represent a conserved mechanism underlying salt stress adaptation across different crops.

Functional enrichment analyses revealed that genes associated with oxidoreductase activity were significantly enriched after 12 h, while genes related to catalytic activity showed extensive enrichment at 24 h (2961 genes), indicating that enzymatic antioxidant defense systems may play a critical role during the prolonged phase of salt stress. In addition, KEGG pathways associated with plant hormone signal transduction, phenylpropanoid biosynthesis, and glutathione metabolism were enriched, further demonstrating that hormonal regulation, ROS scavenging, and secondary metabolic adjustment collectively contribute to plant adaptation under salt stress. These findings are consistent with previous studies in barley [30] under similar salt stress conditions, further supporting the widespread involvement of multi-pathway synergistic regulation in plant salt stress responses.

The identification of 1179 common DEGs across the four time points suggests the existence of a conserved transcriptional regulatory program involved in maintaining long-term salt stress adaptation. WGCNA revealed that the MEblack module exhibited strong correlations with all six physiological parameters, including proline, MDA, H_2_O_2_, SOD, CAT, and POD, highlighting the effectiveness of WGCNA in identifying co-expressed gene networks associated with complex physiological traits. Within this module, *StANN5* and *StANN6* were identified as hub genes based on high module membership (MM ≥ 0.9) and gene significance values (|GS| ≥ 0.9). These genes were co-expressed with other stress-responsive genes, including *UGT75C1*, a UDP-glycosyltransferase involved in secondary metabolic regulation, and *PM27*, a pathogenesis-related protein. It should be noted that WGCNA reveals correlations among gene expression patterns rather than direct regulatory relationships. Therefore, the identification of *StANN5* and *StANN6* as hub genes does not directly demonstrate their regulatory functions, but rather indicates a strong coordination between their expression patterns and salt stress-associated physiological processes. Nevertheless, the presence of annexins among highly connected network nodes is biologically meaningful, as annexins are important calcium-binding proteins involved in Ca^2+^ signal transduction and membrane phospholipid interactions [15]. The coordinated expression of *StANN5* and *StANN6* with genes involved in stress-related pathways, such as phenylpropanoid biosynthesis and glutathione metabolism, suggests that potato annexins may participate in a key regulatory network linking Ca^2+^ signaling regulation with the maintenance of redox homeostasis during salt stress. These findings provide an important foundation for further systematic identification and functional characterization of the annexin gene family in potato. We acknowledge that restricting WGCNA input to consistently differentially expressed genes, while enhancing the specificity of stress-responsive modules, necessarily excludes transiently induced genes such as StANN2 and StANN3. These early responders were therefore analyzed separately through the genome-wide family characterization, ensuring that both sustained adaptive regulators and rapid signaling components were comprehensively represented in our study.

### 4.2. Evolutionary Framework and Structural Conservation of the StANN Family

In this study, nine ANN genes were identified in the potato genome. The size of the StANN gene family is comparable to that reported in other *Solanaceae* species and most dicotyledonous plants, including eight ANN genes in *Arabidopsis*, ten in tomato, and nine in pepper [13,31,32]. The chromosomal distribution of the StANN genes was uneven, with four members clustered on Chr 1, suggesting that this genomic region likely arose through tandem duplication events. Similar chromosomal clustering has been reported in tomato, where *SlANN1*–*SlANN4* are all located on Chr 1, and in mung bean, where *VrANN3*–*VrANN4* are located on chromosome 5 [32,33]. These observations indicate that tandem duplication has likely served as an important evolutionary mechanism driving the expansion of the ANN gene family. Analysis of duplicated gene pairs showed that all Ka/Ks ratios ranged from 0.22 to 0.31, remaining well below 1, indicating that these duplicated genes have been subjected to strong purifying selection during evolution. This finding is consistent with previous reports in *Arabidopsis*, rice, poplar, cotton, and mung bean, in which duplicated ANN paralogs also exhibited Ka/Ks values below 1 [13,19,33]. Such evolutionary conservation suggests that the core biological functions of annexins, particularly their Ca^2+^-dependent phospholipid-binding activity, have been maintained under strong selective constraints. Nevertheless, the high conservation of coding sequences does not preclude functional divergence among duplicated genes. Instead, duplicated paralogs may undergo subfunctionalization through regulatory diversification, including changes in promoter architecture and expression patterns, thereby facilitating their long-term retention within the genome [34]. Consistent with this hypothesis, distinct expression profiles of StANN genes were observed across different tissues and at multiple time points during salt stress treatment (Figure 6A), supporting functional differentiation among family members.

Structural analysis demonstrated that all StANN proteins retained the characteristic annexin domains, with the conserved motifs 1, 2, 3, 5, and 6 present in every family member, reflecting the highly conserved structural framework of annexins in land plants [13]. However, variation was observed in gene structure, with StANN genes containing four to six exons and exhibiting substantial differences in intron length. Notably, considerable structural divergence was detected even between the closely related paralogs *StANN1* and *StANN4*. These findings suggest that dynamic changes in intron architecture may have contributed to gene family diversification while preserving protein-coding sequences, a pattern that has also been reported for ANN gene families in maize and cotton [35,36]. In addition, several lineage-specific motifs imply potential functional specialization among individual StANN proteins. For example, Motif 9 was exclusively identified in *StANN1*, *StANN3*, and *StANN4*, whereas Motif 10 was restricted to *StANN3* and *StANN4*. These unique structural features may confer distinct regulatory functions on specific family members, although their precise biological roles remain to be elucidated through structural characterization and functional validation.

### 4.3. Expression Dynamics and Functional Implications of StANN Genes

The differential expression patterns of StANN genes across tissues suggest that individual family members may participate in distinct physiological processes. Among them, *StANN8* exhibited a pronounced root-preferential expression pattern (Z-score = 2.23), which is consistent with the root-enriched expression of *OsANN1* in rice and *GhANN1* in cotton. Previous studies have demonstrated that both *OsANN1* and *GhANN1* facilitate salt tolerance by regulating Ca^2+^ influx into root cells, thereby activating the SOS-dependent Na^+^ extrusion pathway [16,19]. However, it should be noted that the transcriptomic dataset analyzed in this study was primarily derived from leaf tissues. Therefore, the expression dynamics and biological functions of StANN genes in roots, stems, and other tissues, particularly their potential roles in salt ion uptake and long-distance stress signaling, require further investigation. In contrast, *StANN1*, *StANN4*, and *StANN9* were predominantly expressed in floral tissues, suggesting potential functions in reproductive development. This observation is consistent with the reported role of *AtANN5* in regulating pollen development in *Arabidopsis* [37]. Notably, no detectable expression of *StANN1*, *StANN8*, or *StANN9* was observed in leaves under either control or salt stress conditions (Figure 6B, marked as “X”), indicating that these genes are unlikely to play major roles in leaf-mediated salt stress responses but may instead function in specific tissues or developmental stages.

Under salt stress, StANN genes displayed distinct temporal expression patterns, indicating functional specialization during different phases of the stress response. *StANN5* and *StANN6* showed sustained upregulation from 3 to 48 h after salt treatment, representing a typical long-term induction pattern. Similar expression profiles have been reported for *GhANN1*, whose induction under salinity is associated with enhanced ABA biosynthesis and ROS scavenging [19]. Likewise, *OsANN3* has been shown to promote ABA-dependent stomatal closure, thereby improving stress adaptation [17]. Consistent with these findings, the promoters of *StANN5* and *StANN6* are enriched in ABRE cis-regulatory elements, suggesting that their salt-induced expression may be regulated through ABA signaling. Nevertheless, the contribution of individual cis-elements to their transcriptional regulation remains to be experimentally validated through promoter activity assays and transcription factor binding analyses. Unlike the sustained induction of *StANN5* and *StANN6*, *StANN2* and *StANN3* were rapidly induced at 3 h after salt treatment, followed by a gradual decline in transcript abundance, representing a transient early-response pattern. This expression profile resembles that of *AtANN1*, which functions in the early phase of salt stress by mediating rapid Ca^2+^ signaling and stress perception [15]. These observations suggest that *StANN2* and *StANN3* may primarily participate in early salt stress signaling, whereas *StANN5* and *StANN6* are more likely involved in sustained adaptive responses during prolonged stress exposure. Such functional partitioning between “early signaling responders” and “long-term stress regulators” may represent a conserved strategy by which plant annexins coordinate abiotic stress adaptation, consistent with the regulatory mechanism proposed for tomato *AnnSp2* [18].

Promoter analysis further revealed that most StANN genes contain abundant ABRE and other ABA-responsive cis-elements, supporting the involvement of ABA signaling in regulating their expression under salt stress. This finding agrees with previous studies demonstrating that annexins participate in ABA-mediated stress responses. For example, overexpression of tomato *AnnSp2* increases endogenous ABA accumulation [18], whereas *GhANN1* directly contributes to ABA biosynthesis under salt stress [19]. In addition, the presence of MBS (MYB-binding site) elements suggests potential crosstalk between annexin-mediated Ca^2+^ signaling and MYB-dependent transcriptional regulation. This possibility is supported by the interaction between *GhANN1* and *GhWRKY40-like*, which coordinately regulate stress responses in cotton [19]. Collectively, these observations suggest that potato annexins may function as important regulatory hubs integrating Ca^2+^ signaling, hormone-mediated regulation, and redox homeostasis during salt stress.

Although the salt tolerance functions of individual StANN genes remain to be genetically validated, the integration of expression profiling, promoter cis-element analysis, and co-expression network analysis enabled the identification of several promising candidate genes. In particular, *StANN5* and *StANN6*, characterized by sustained salt-induced expression, abundant stress-responsive cis-elements, and high connectivity within the co-expression network, are likely to play central roles in long-term salt stress adaptation. In contrast, *StANN2* and *StANN3* may function primarily in the perception and transmission of early salt stress signals. These findings provide new insights into the molecular mechanisms by which annexins contribute to salt stress adaptation in potato and establish a theoretical foundation for future functional characterization of StANN genes and the molecular breeding of salt-tolerant potato cultivars.

## 5. Conclusions

This study provides a comprehensive characterization of the annexin gene family in potato and elucidates its involvement in salt stress responses through an integrated transcriptomic and network-based approach. We identified nine StANN genes that share conserved structural features with annexins from other plant species but exhibit distinct expression dynamics under salinity. WGCNA-based co-expression analysis positioned annexin genes as hub components within a salt-responsive regulatory network closely associated with redox homeostasis and stress signaling. The differential temporal expression patterns observed among StANN members—specifically the sustained upregulation of *StANN5* and *StANN6* versus the early transient response of *StANN2* and *StANN3*—suggest divergent temporal roles in the salt stress response trajectory, potentially reflecting a division between sustained adaptive regulators and rapid initial responders. The prevalence of ABA-responsive (ABRE) and dehydration-responsive (DRE/CRT) cis-elements in StANN promoters further supports their integration into hormone-mediated stress signaling pathways. While our transcriptomic and bioinformatic analyses establish strong correlative evidence for annexin-mediated salt tolerance, functional validation through transgenic or gene-editing approaches—particularly targeting *StANN5* and *StANN6* for their sustained induction, and *StANN2* and *StANN3* for their early responsiveness—will be essential to confirm their causal roles and translate these findings into practical breeding strategies.

## Figures and Tables

**Table 1:** Characterization of putative potato annexin proteins.

Gene ID	Protein Symbol	AA	Mw (kD)	pI	Instability Index	Aliphatic Index	GRAVY	Localization Predicted by WoLF PSORT
DMG400040554	StANN1	325	36.63	5.34	33.51	101.38	−0.214	cyto, cysk, nucl
DMG400019446	StANN2	317	36.33	5.4	35.04	90.6	−0.541	cyto, plas, chlo, nucl, mito
DMG402019427	StANN3	314	35.85	5.37	43.2	95.41	−0.511	cyto, cyto_E.R, chlo, nucl
DMG401019427	StANN4	316	36.38	6.18	46.39	95.09	−0.375	cysk, nucl, cyto, cyto_E.R.
DMG400001879	StANN5	318	35.75	6.36	37.69	95.97	−0.235	nucl, cyto_nucl, pero, cyto
DMG400017714	StANN6	318	35.69	6.47	36.49	94.69	−0.196	nucl, chlo, cyto
DMG400007966	StANN7	316	35.59	9.07	31.74	91.2	−0.305	chlo, cyto, nucl, mito, plas
DMG400021817	StANN8	315	35.87	5.35	31.46	91.71	−0.39	chlo, nucl, cyto, mito, plas
DMG400007482	StANN9	309	35.76	5.97	34.17	91.23	−0.536	cyto, cysk

chlo—chloroplast; cyto—cytoplasm; cyto_E.R.—cytoplasm/membrane of endoplasmatic reticulum; cysk—cytoskeleton; mito—mitochondria; nucl—nucleus; plas—plastids. *StANN5* and *StANN6* correspond to *ANN-1* and *ANN-2* in Figure 2E,F, respectively.

## Data Availability

Data will be made available on request.

## Supplementary Materials

The following supporting information can be downloaded at: https://www.mdpi.com/article/10.3390/genes17091094/s1, Figure S1: Correlation analysis of transcriptome sequencing samples under drought and rewatering treatment and PCA analysis; Figure S2: Correlation analysis of RNA-seq and qRT-PCR; Figure S3: Physiological responses of potato plants to 150 mM NaCl stress; Figure S4: Collinear relationships of the StANN family genes; Table S1: Specific primers for qRT-PCR; Table S2: Statistics of transcriptome sequencing data; Table S3: Sequencing data alignment and distribution statistics of reads in different regions; Table S4: Hub genes in responses to salt in potato; Table S5: Gene duplication and Ka/Ks analysis; Table S6: Conserved motifs of StANN Family proteins; Table S7: Normality and variance homogeneity tests for physiological data.
