# Supplementary material for: Genome-Wide Identification and Characterization of the Annexin (ANN) Gene Family in Potato (Solanum tuberosum L.) and Its Role in Salt Stress Response Revealed by Integrated Transcriptome and WGCNA Analysis
Source: Genes (Basel). 2026 Sep 11;17(9):1094. doi: 10.3390/genes17091094 (PMC13606461; doi:10.3390/genes17091094)
Supplement: Supplementary file 1 [file genes-17-01094-s001.zip › genes-4486081-supplementary.pdf]

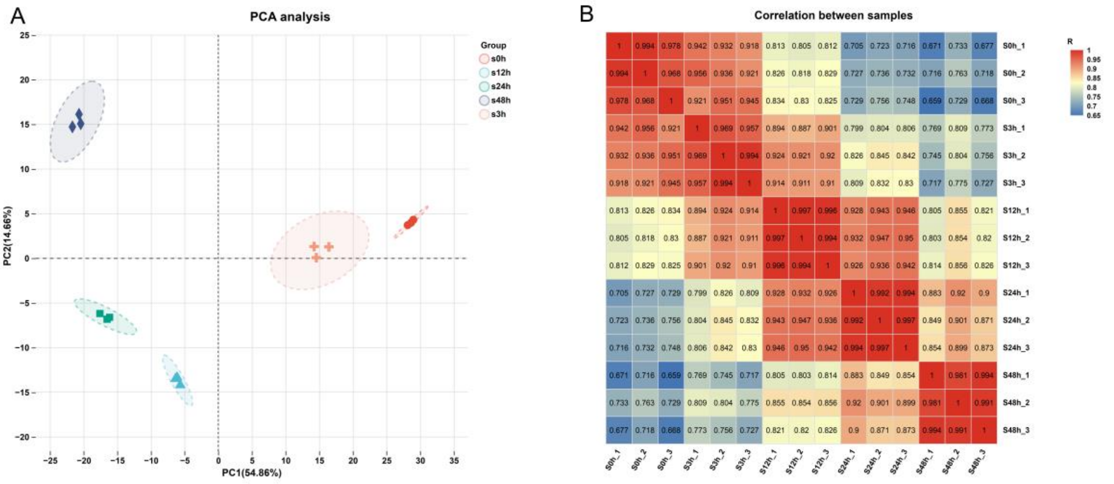

Figure S1. Correlation analysis of transcriptome sequencing samples under drought and rewatering treatment (a) and PCA analysis (b). These two figures demonstrate that the sampling of our transcriptome sequencing samples is reasonable.

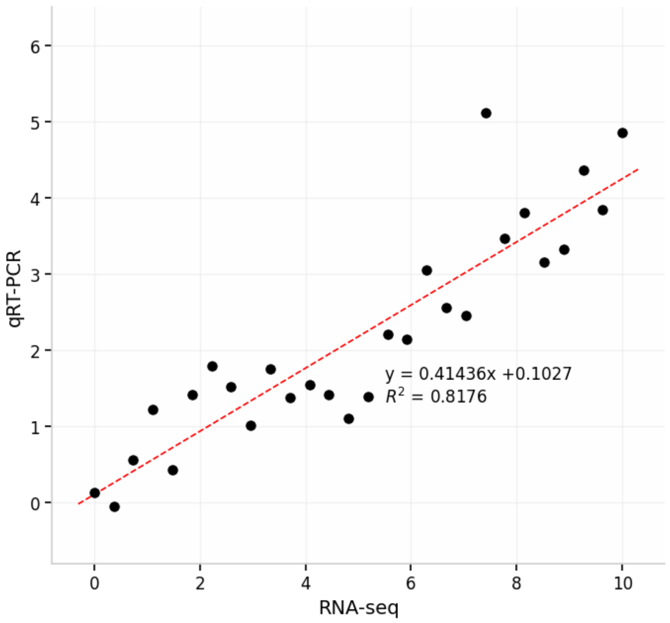

Figure S2. Correlation analysis of RNA-seq and qRT-PCR.

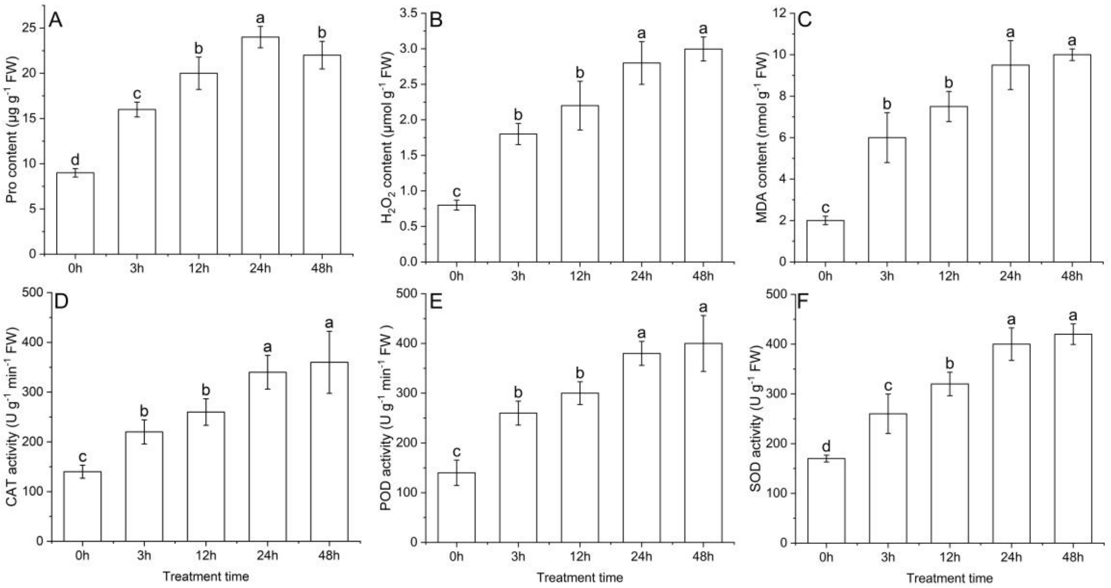

Figure S3. Physiological responses of potato plants to 150 mM NaCl stress. (A) Proline content, (B) H_2O_2 content, (C) MDA content, (D) CAT activity, (E) POD activity, and (F) SOD activity in potato leaves at 0, 3, 12, 24, and 48 h after salt treatment. Different lowercase letters indicate statistically significant differences among time points at $P < 0.05$ (one-way ANOVA followed by Duncan's multiple range test). Values represent the mean ± SD (n = 3) of three independent biological replicates.

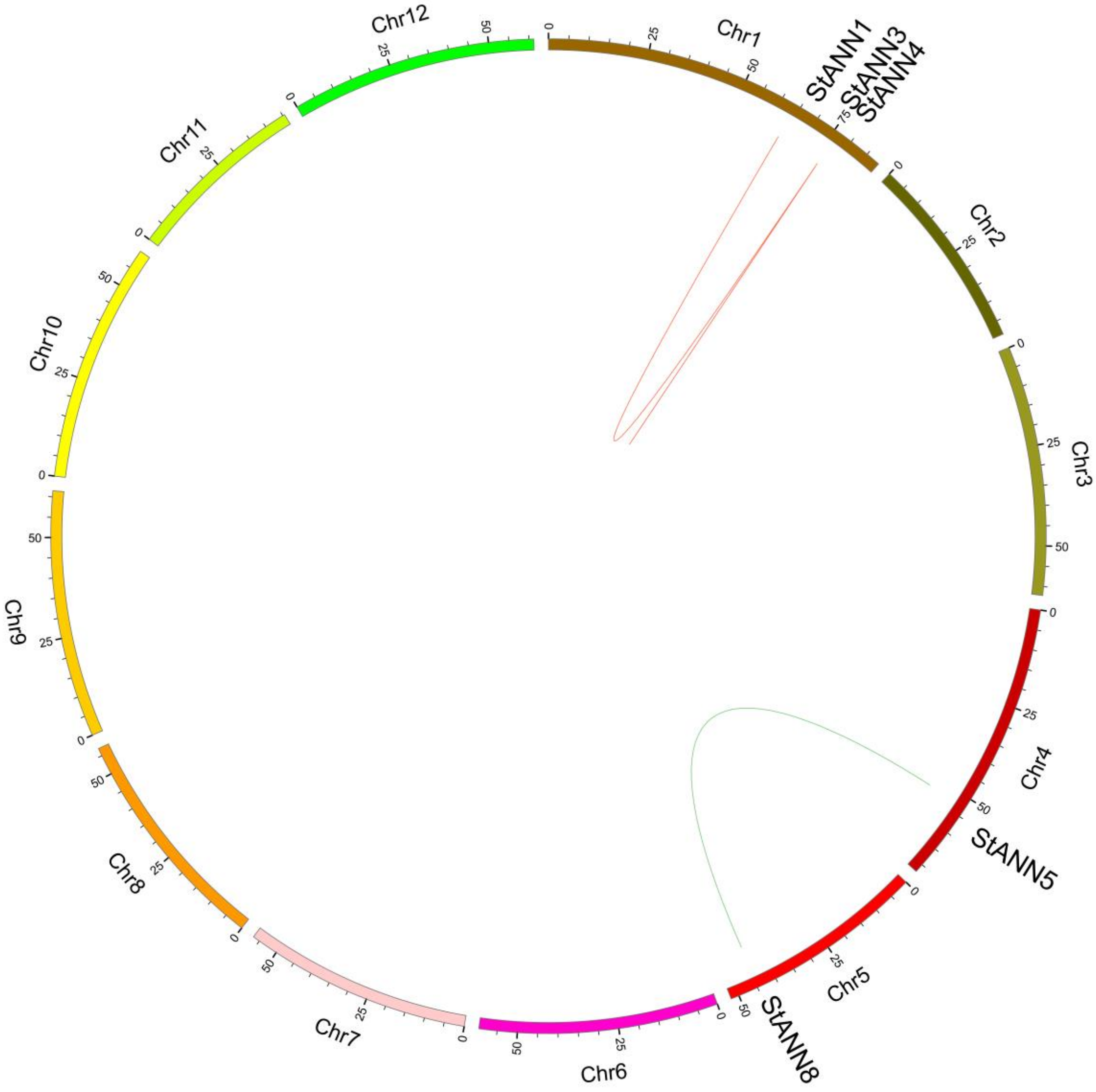

Figure S4. Collinear relationships of the StANN family genes.

Table S1. Specific primers for qRT-PCR.

Gene ID	Forward primer (3′–5′)	Reverse primer (3′–5′)
EF1A	TCACATCAACATTGTGGTTATTGG	TTAAGCTGGTCAAGAGAGCCTCAAG
StANN2	CTCCATTGACCAGGACATGAAGAG	TTTGGCACCAAGCAGTGTCATGAG
StANN3	AGCTCCATCTTAAGGCCATC	GTAACTATGACTCGGGTCACTG
StANN5	GTGCAACGTTCAACCACTAC	CCCAATCAAAGCCAGAAG
StANN6	TTAGCCACAAGGAGCAAAGC	TCCCTCTTCTGGTACTCGTTAG
DMG400001474 (SLC38A2)	CAGCCAGTAGTAGATGACCA	TTGTGGTGGTGACTTGTGG
DMG400021966 (LAC14)	AACAAATGTAGAGATTGTGTT	AATTCATCTGTTGGTCAGAA
DMG400024120 (SLC15A3)	ATGGTGAACCTGCGGTGCGT	GGTAAAGGAGGAGGTCATCC
DMG400031454 (UGT9)	ATGGATACTACTACTAGTTT	CATGAAGTCTTGGGTTTAC
DMG402005726 (CPK10)	ATGCATCCTTCCTTGATGTT	GTAGATGAACTCTGTCAAT
DMG400014594 (ERF1)	ATGTCAAGCAACTCAAGCCC	GATAGCCTCAGCAGCCCTT

Table S2. Statistics of transcriptome sequencing data.

Sample	Clean reads	Clean bases	Error rate(%)	Q20(%)	Q30(%)	GC content(%)
S0h_1	39,740,482	5,949,711,185	0.0117	98.63	96.90	42.48
S0h_2	39,084,302	5,848,861,416	0.0118	98.58	96.79	42.46
S0h_3	43,930,722	6,577,024,827	0.0118	98.55	96.74	42.20
S3h_1	46,620,368	6,976,006,723	0.0122	98.25	96.03	42.42
S3h_2	45,526,382	6,813,287,799	0.0117	98.59	96.82	42.26
S3h_3	41,530,746	6,214,920,959	0.0117	98.60	96.83	42.24
S12h_1	45,889,778	6,868,958,441	0.0118	98.58	96.80	42.13
S12h_2	43,716,472	6,543,292,433	0.0116	98.69	97.00	42.15
S12h_3	48,275,012	7,222,742,566	0.0116	98.67	96.98	42.23
S24h_1	37,730,052	5,645,322,849	0.0116	98.69	97.03	42.23
S24h_2	49,124,030	7,349,299,779	0.0117	98.61	96.84	42.20
S24h_3	51,214,464	7,663,874,193	0.0117	98.64	96.92	42.20
S48h_1	44,168,692	6,602,073,063	0.0117	98.61	96.87	42.51
S48h_2	42,350,482	6,332,320,923	0.0117	98.61	96.87	42.28
S48h_3	45,340,456	6,781,265,968	0.0119	98.50	96.63	42.42

Table S3. Sequencing data alignment and distribution statistics of reads in different regions.

Sample	Total reads	Total mapped	Multiple mapped	Uniquely mapped
S0h_1	39,740,482	33,885,151(85.27%)	1,246,141(3.14%)	32,639,010(82.13%)
S0h_2	39,084,302	33,217,795(84.99%)	1,292,633(3.31%)	31,925,162(81.68%)
S0h_3	43,930,722	37,233,480(84.75%)	1,281,041(2.92%)	35,952,439(81.84%)
S3h_1	46,620,368	39,331,949(84.37%)	1,598,987(3.43%)	37,732,962(80.94%)
S3h_2	45,526,382	38,571,725(84.72%)	1,466,012(3.22%)	37,105,713(81.5%)
S3h_3	41,530,746	35,215,352(84.79%)	1,316,388(3.17%)	33,898,964(81.62%)
S12h_1	45,889,778	38,760,251(84.46%)	1,564,299(3.41%)	37,195,952(81.05%)
S12h_2	43,716,472	37,060,596(84.77%)	1,484,392(3.4%)	35,576,204(81.38%)
S12h_3	48,275,012	40,930,326(84.79%)	1,693,365(3.51%)	39,236,961(81.28%)
S24h_1	37,730,052	32,120,353(85.13%)	1,307,226(3.46%)	30,813,127(81.67%)
S24h_2	49,124,030	41,726,771(84.94%)	1,610,836(3.28%)	40,115,935(81.66%)
S24h_3	51,214,464	43,574,190(85.08%)	1,706,795(3.33%)	41,867,395(81.75%)
S48h_1	44,168,692	37,086,254(83.97%)	2,085,235(4.72%)	35,001,019(79.24%)
S48h_2	42,350,482	35,703,861(84.31%)	1,707,933(4.03%)	33,995,928(80.27%)
S48h_3	45,340,456	38,137,155(84.11%)	2,035,709(4.49%)	36,101,446(79.62%)

Table S4. Hub genes in responses to salt in potato.

ID	ImConn_black	Gene name	Gene Description
DMG401001531	217.21	TBL34	Conserved gene of unknown function
DMG400010770	215.97	grxC	Glutaredoxin
DMG400001474	211.32	SLC38A2	Amino acid transporter
DMG400021895	210.2	WRKY33	WRKY33
DMG400021966	210.05	LAC14	Laccase
DMG400000731	209.77	RD2	Response to desiccation RD2
DMG400001879	209.71	ANN-1(StANN5)	Annexin p35
DMG400009527	209.7	SGT1	UDP-xylose phenolic glycosyltransferase
DMG400014594	208.96	ERF1	Ethylene response factor
DMG400031067	208.23	-	Conserved gene of unknown function
DMG400024120	208.42	SLC15A3_4	Peptide transporter
DMG400021116	207.67	FG2	UDP-glucoronosyl/UDP-glucosyl transferase family protein
DMG400004899	207.22	GPT	Alanine aminotransferase
DMG400002826	204.73	FBK15	Kelch repeat-containing F-box family protein
DMG400023957	204.32	TYRAAT	Prephenate dehydrogenase
DMG400017714	203.99	ANN-2(StANN6)	annexin p34-like protein
DMG400027043	203.91	SLC25A3	Mitochondrial phosphate carrier protein
DMG400029544	203.86	RNF6	Ring finger protein
DMG400015678	203.52	10HGO	10-hydroxygeraniol oxidoreductase
DMG400020541	203.1	PPM1L	Protein phosphatase 2C
DMG401005047	203.04	RPK	Receptor protein kinase
DMG400031454	202.42	UGT9	UDP-glucose:glucosyltransferase
DMG400006737	201.93	UGT75C1	Cold-induced glucosyltransferase
DMG400008267	201.6	CYP72A54	CYP72A54
DMG400001528	201.6	CHIB	Class II chitinase
DMG400005456	201.54	PDP	Protein phosphatase 2c
DMG402005726	201.29	CRK10	Serine-threonine protein kinase, plant-type
DMG400009525	201.13	SGT1-1	UDP-glucose:glucosyltransferase
DMG400007613	200.7	AOX1	Alternative oxidase
DMG400024857	200.3	SKP2	F-box protein SKP2A
DMG400009528	199.68	SGT1-2	UDP-xylose phenolic glycosyltransferase
DMG400030633	199.41	HACL1	Pyruvate decarboxylase
DMG400000599	198.67	bHLH47	DNA-binding protein
DMG400009095	198.59	ABCC2	abc-transoprter
DMG400024661	198.42	BRI1	Brassinosteroid LRR receptor kinase
DMG400007141	198.22	ABCF2	ABC transporter family protein
DMG400006708	198.05	CXE20	HSR203J protein
DMG400010763	197.49	grxC-1	Glutaredoxin
DMG402002771	197.46	BZIP1S	Anaerobic basic leucine zipper protein

DMG400001715	196.62	PUP11	ATPUP11
DMG400031519	196.28	CYP83B1-like	CYP83B1-like
DMG400008739	195.8	SLC47A	Mate efflux family protein
DMG400031411	192.37	PM27	Seed maturation protein PM27
DMG400010131	192.31	KTI5	Nb cell death marker
DMG400027283	192.28	HSFF	AT-HSFB3
DMG402018740	191.88	TCH4	Xyloglucan endotransglucosylase/hydrolase 9
DMG400015681	191.8	TMVRN	Resistance gene
DMG400010300	191.66	CFL	Actin-depolymerizing
DMG401027676	191.62	MCU	MCU
DMG400030503	191.56	-	Conserved gene of unknown function

Table S5. Gene duplication and Ka/Ks analysis.

Sequence	Duplication type	Ka	Ks	Ka/Ks
StANN1&StANN3	Proximal	0.174329	0.782741	0.222716
StANN1&StANN4	Proximal	0.184824	0.805795	0.229369
StANN3&StANN4	Tandem	0.056678	0.222034	0.255267
StANN5&StANN8	Segmental	0.21901	0.696666	0.314368

Table S6. Conserved motifs of StANN family proteins.

Motif	length (aa)	Amino acid conserved sequence
Motif1	50	KAFKGLGTBEKAIISILGHRNASQRKLIRZTYQQLYNEDLLKDLDSELSG
Motif2	50	IWCJKSPEKYFAEVLRAAIKGLGTDEDSLTRVIVTRAEVDMKKIKEEYYK
Motif3	41	QVIVEIACARSPDDLVAVRQAYHALFKKSLEEDIAYHVSGP
Motif4	50	EAAILHEKISDKQYDDEEIIRILSTRSKAQLKATFNHYKDNYGFSIDKDL
Motif5	26	DFEKAVLLWTYDPAERDARLANEALK
Motif6	28	RNSTTLDSAIADDTSGDYRKFLLTLLGA
Motif7	21	VRKLLIALVSSYRYEGEEVDP
Motif8	21	MASLRIPDVVPSPAEDAETLR
Motif9	14	SCGKGLLESILKVV
Motif10	6	KKTITQ

Table S7. Normality and Variance Homogeneity Tests for Physiological Data.

Parameters	F value	P value	Shapiro-Wilk P	Levene P
H_2O_2	43.991	2.58×10^{-6}	0.8928	0.5598
MDA	44.578	2.42×10^{-6}	0.9921	0.8376
Proline	100.862	4.87×10^{-8}	0.3068	0.832
CAT	18.616	1.26×10^{-4}	0.94	0.5068
SOD	43.128	2.83×10^{-6}	0.2597	0.7496
POD	32.745	1.01×10^{-5}	0.9433	0.4744
